# Regression equation analysis enhances detection of conduction slowing beyond axonal loss in diabetic neuropathy

**DOI:** 10.1016/j.heliyon.2024.e39712

**Published:** 2024-10-22

**Authors:** Nizar Souayah, Zhao Zhong Chong, Tejas Patel, Abu Nasar, Ankit Pahwa, Howard W Sander

**Affiliations:** a Department of Neurology, Rutgers University New Jersey Medical School, 90 Bergen Street DOC 8100, Newark, NJ, 07101, USA; b SMR Consulting, 407 Elmwood Avenue, Sharon Hill, PA 19079, USA; c Department of Neurology, NYU Grossman School of Medicine, New York, NY 10016, USA

**Keywords:** Regression analysis, Diabetic neuropathy, Amyotrophic lateral sclerosis, Conduction velocity slowing, Demyelination

## Abstract

**Objectives:**

To evaluate the utility of regression analysis in the assessment of conduction slowing in diabetic distal symmetrical polyneuropathy (DSP) and in identifying superimposed demyelination beyond fiber loss.

**Background:**

Causes of conduction slowing beyond pure axonal loss has been attributed to an additional demyelinating component. We therefore evaluated the utility of regression analysis in the assessment of conduction slowing in diabetic DSP and in identifying superimposed demyelination beyond fiber loss.

**Methods:**

We previously established regression analysis to develop confidence intervals that assess the range of conduction slowing from primary demyelination in patients with chronic inflammatory demyelinating polyneuropathy (CIDP). In this study, by using the regression equations, we analyzed conduction slowing in patients with diabetic DSP.

**Results:**

Mean conduction velocity (CV) was significantly slower in diabetic DSP than in the non-diabetic DSP for all tested nerves. More patients were found to fulfill the regression equation criteria in the diabetic group compared to the non-diabetic group (47.0 % vs. 23.3 %). The estimated likelihood of having more than two motor nerves with CV slowing in the demyelination range by American Academy of Neurology or regression equations criteria was significantly higher in the diabetic DSP (0.73) compared to non-diabetic DSP (0.52).

**Conclusions:**

Conduction slowing in diabetic DSP beyond what is expected exclusively from axonal loss could be identified by regression analysis.

## 1 Introduction

Diabetic neuropathy (DN) is the most common chronic complication of diabetes with a prevalence that widely ranges from 8.3 % to 79 %, and is a common cause of limb amputation [[1], [2], [3], [4], [5]]. Distal symmetrical polyneuropathy (DSP) is the most common form of diabetic neuropathy. The initial studies implied that diabetic DSP is an irreversible axonal neuropathy with axonal degeneration, regeneration, and a lesser degree of secondary demyelination without axonal atrophy [[6], [7], [8]]. However, it is increasingly recognized that the conduction velocity (CV) slowing in diabetic DSP exceeds that observed in non-diabetic distal axonal neuropathies [[9], [10], [11]]. Early axonal loss and delay of immunotherapy initiation in CIDP were associated with long-term nerve fiber loss and disability [12]. Evidence of conduction slowing was associated with worse glycemic control in diabetes [13]. In a prospective study, immunoglobin injection significantly relieved pain in painful diabetic DSP [14]. These findings suggest that peripheral demyelination may occur early before the axonal loss.

Although axonal loss is considered the primary pathological damage in diabetic DSP, conduction slowing beyond what is expected by decreased compound muscle action potential (CMAP) has been reported [11]. Furthermore, severe proximal nerve root demyelination without axonal loss has been observed in autopsies of patients with diabetic DSP [15]. Demyelination superimposed on axonal loss was proposed to explain CMAP amplitude-independent motor nerve conduction slowing in diabetic DSP [11]. In addition, the reduction in nerve conduction velocity always precedes the reduction of CMAP amplitude in DSP. Baba's classification has been commonly used in Japan for nerve conduction study, which has been proven very effective in evaluating dysfunctions of large nerve fibers [16]. However, clinical examination, as well as conventional nerve conduction study criteria, do not permit a clear distinction between pure loss of large fibers and loss of large fibers with accompanying minor degrees of demyelination. Conduction slowing due to superimposed mild demyelination in diabetic neuropathy is unlikely to be recognized as such until it reaches a severity that is well outside the usual spectrum of pure axonal loss. This is because of the alternative explanation related to the loss of fast conducting fibers due to axonopathy. Since peripheral nerve demyelination is potentially reversible, there may be a benefit from new means of identifying diabetic patients with this type of neuropathy.

Detecting demyelination in diabetic neuropathy holds significant clinical importance, as it enables early identification and management of demyelinating components that may coexist with axonal damage. Identifying this pathology allows for targeted therapeutic interventions, potentially slowing disease progression and improving patient outcomes. If demyelination is predicted using the proposed regression equation, treatment options could include immunotherapy to enhance nerve function. These interventions, when implemented promptly, may mitigate the impact of demyelination, underscoring the value of accurate detection methods in diabetic neuropathy management.

The objective of this study is to evaluate the utility of regression analysis in the assessment of conduction slowing in diabetic DSP. Previously, using a well-defined population of patients with chronic inflammatory demyelinating polyneuropathy (CIDP) [17], we determined the range of motor nerve conduction variation and developed regression equations and confidence limits for motor nerve conduction slowing related to primarily acquired demyelination [18]. In this study, we analyzed groups of patients with diabetic and non-diabetic DSP to look for motor nerve conduction slowing in the range predicted by the regression equations to further characterize the distribution of conduction slowing in diabetic DSP, as well as to display the limits of conduction slowing, beyond which the diagnosis of primary axonopathy is questionable.

## 2 Materials and methods

### 2.1 Subjects

The study was conducted retrospectively using neurophysiological data obtained from 219 patients diagnosed with diabetic DSP, 219 patients with non-diabetic axonal DSP and 95 patients with ALS. The diagnosis of diabetic DSP was defined based on known history of diabetes mellitus, the presence of symmetric signs of distal weakness and sensory dysfunction, and the absence or reduction of deep tendon reflexes. The diagnosis of axonal sensorimotor polyneuropathy is based on the absence of a history of diabetes, hyporeflexia or areflexia, the presence of distal weakness and sensory dysfunction, and the absence of electrodiagnostic research criteria for CIDP as per the American Academy of Neurology (AAN) [17]. Electrodiagnostic testing was performed as previously described [18]. Nerve conduction study parameters, including CMAP amplitude, distal latency (DL), conduction velocity (CV), and F latency of the median, ulnar, tibial, and fibular nerves, were collected.

For diabetic DSP, exclusion criteria include the absence of clinical evidence of neuropathy, the presence or co-existence of other known causes of neuropathy (e.g., B12 deficiency, exposure to neurotoxic agents), use of anti-inflammatory drugs, presence of malignancy, or other causes of systemic inflammation and active infection, or with co-existence of HIV or other blood contagious diseases. The DSP cases without presence of diabetes mellitus were included in the non-diabetic DSP group.

### 2.2 Regression analysis

As previously described [18], we used electrodiagnostic data from CIDP patients, performed regression analysis to develop confidence intervals that assessed the range of conduction slowing from primary demyelination. Briefly, we retrospectively identified a group of 76 patients with CIDP using the AAN criteria for primary demyelination [17]. Then, we used data from a second group of 38 well-defined CIDP patients to validate the developed equations [18].

In this study, we used the developed equations to assess conduction slowing in the two study groups: diabetic DSP (219 patients) and non-diabetic axonal DSP (219 patients). The data from the ALS group (95 patients) demonstrated in our previous study [18] was also compared.

### 2.3 Statistical analysis

The developed equations [18] were utilized to measure the range of conduction slowing in DSP groups. Basic data summary statistics were performed using MS Excel 2016. SAS Software (version 9.4) was used to perform advanced statistical analysis of the data. Categorical variables were summarized by their counts and percentages, and the distribution of groups was compared using Fisher's exact test or chi-square test as appropriate. Continuous variables were descriptively summarized by their means and standard deviations (mean and SD) and the differences between the group means were tested using a non-parametric Mann-Whitney *U* test.

Linear regression analysis was used to identify the dependence of CV, DL and F on CMAP amplitude. Transformed CMAP amplitude was used as a dependent variable whereas transformed CV, DL or F was used as an independent variable.

Pearson's correlation coefficient (r) was used to evaluate the correlation between CMAP amplitude and CV, DL, and F. Interpretation of the amplitude of correlation coefficients was based on Cohen's conventions categorizing it as either small/weak, medium/moderate, or large/strong correlation (Cohen 1988):

• 0.1–0.3: Small/Weak
• 0.3–0.5: Medium/Moderate
• > 0.5: Large/Strong

The relationship between transformed CMAP amplitude and transformed DL, CV, and F were compared both within and between the studied groups using scatterplots, linear regression analysis, and analysis of residuals [11]. Multiple linear regression analysis models were designed to compare the Y-intercepts (a representation of CV, DL, or F) and the slopes of the regression lines (a measure of the relationship between CMAP amplitude and CV, DL, or F) between the groups for both upper and lower extremities.

Categorical variables were summarized by their counts and percentages, and the distribution of groups was compared using chi-square test and Fisher's exact tests. A Z-test of proportions was used to compare the two proportions. Continuous variables were summarized by their means and standard deviations along with ranges, and the distribution of groups was tested using *t*-test and analysis of variance. All tests for statistical significance were two-sided with a significance level of 0.05.

## 3 Results

The demographic and neurophysiological data of diabetic DSP, non-diabetic DSP, and ALS is summarized in Table 1. There is no significant difference between the mean age distribution of patients between the three study groups (Table 1).

**Table 1:** Demographic and neurophysiological profile of diabetic DSP, non-diabetic DSP and ALS groups.

Parameter/Groups	Diabetic DSP (DDSP) (N = 219)		Non-Diabetic DSP (NDDSP) (N = 219)	ALS (N = 95)
	Values	*P*-value		
**Age (Years), Mean ± SD**	59.6 ± 12.20	>0.05	58.4 ± 14.00	58.5 ± 11.76
**Gender (% Male)**	42.9 %	<0.01 *vs.* NDDSP or ALS	49.8 %	61.1 %
**Distal Latency (ms), Mean ± SD**				
Median	4.7 ± 1.55	<0.01 *vs.* NDDSP; <0.05 *vs.* ALS	4.1 ± 1.15	4.4 ± 1.07
Ulnar	3.3 ± 0.92	<0.05 *vs.* NDDSP; <0.01 *vs.* ALS	3.1 ± 0.73	3.5 ± 1.11
Tibial	5.5 ± 1.40	>0.05 *vs.* NDDSP; <0.05 *vs.* ALS	5.4 ± 1.73	5.7 ± 1.82
Fibular	4.9 ± 1.58	>0.05 *vs.* NDDSP; <0.05 *vs.* ALS	4.7 ± 1.18	5.2 ± 1.29
**CMAP amplitude (mV), Mean ± SD**				
Median	6.6 ± 2.95	<0.05 *vs.* NDDSP; <0.01 *vs.* ALS	6.8 ± 2.99	4.0 ± 3.28
Ulnar	6.8 ± 3.01	<0.01 *vs.* NDDSP or *vs.* ALS	7.4 ± 2.91	4.7 ± 2.94
Tibial	4.2 ± 3.16	<0.01 *vs.* NDDSP or *vs.* ALS	5.0 ± 3.17	4.9 ± 3.21
Fibular	3.4 ± 2.18	<0.05 *vs.* NDDSP; <0.01 *vs.* ALS	3.6 ± 2.25	3.0 ± 2.33
**Conduction velocity (m/s), Mean ± SD**				
Median	46.1 ± 7.44	<0.05 *vs.* NDDSP; <0.01 *vs.* ALS	49.5 ± 6.14	50.2 ± 8.15
Ulnar	48.2 ± 8.14	<0.01 *vs.* NDDSP; <0.01 *vs.* ALS	52.2 ± 7.63	53.1 ± 9.02
Tibial	38.1 ± 7.84	<0.05 *vs.* NDDSP; <0.01 *vs.* ALS	40.7 ± 5.90	41.6 ± 5.84
Fibular	40.0 ± 7.45	<0.05 *vs.* NDDSP; <0.05 *vs.* ALS	42.8 ± 6.78	43.0 ± 5.65
**F response (converted percentages), Mean ± SD**				
Median	108.5 ± 16.86	>0.05 *vs.* NDDSP or ALS	106.6 ± 17.98	107.7 ± 30.02
Ulnar	102.6 ± 16.54	<0.05 *vs.* NDDSP; <0.01 *vs.* ALS	99.5 ± 11.68	97.1 ± 10.21
Tibial	95.3 ± 18.30	>0.05 *vs.* NDDSP *or* ALS	95.3 ± 14.72	94.1 ± 10.24
Fibular	91.6 ± 17.11	>0.05 *vs.* NDDSP or ALS	88.6 ± 15.28	88.3 ± 11.31

The confidence intervals derived from the regression equations based on CIDP patients were previously developed and validated, and the equations were applied in this study to DSP patients [18]. As shown in Table 1, the mean CV in all nerves tested was significantly lower among the diabetic DSP group compared to that of the ALS and non-diabetic DSP groups for all four tested nerves. In addition, mean F latency was significantly prolonged in the diabetic DSP group compared to the ALS and non-diabetic groups for the ulnar nerve. In addition, the CMAP amplitude was significantly higher in the diabetic DSP group compared to the ALS group for median, ulnar, and fibular nerves (Table 1).

The proportion of motor nerves with a CV below 80 % and 70 % of the lower limit of normal was significantly higher in the diabetic DSP group (19.9 % and 13.2 %) compared to the non-diabetic DSP group (9.0 % and 6.7 %) and the ALS group (8.0 % and 7.4 %, *P* < 0.0001, Table 2). However, the percent of motor nerves with CMAP amplitude below the lower limit of normal was significantly lower in the diabetic DSP (36.2 %) and non-diabetic DSP (29.7 %) groups compared to the ALS group (50.9 %, *P* < 0.0001, Table 2).

**Table 2:** Nerve conduction studies of diabetic DSP, non-diabetic DSP and ALS groups.

Parameters	Diabetic DSP (DDSP) (N = 219)		Non-Diabetic DSP (NDDSP) (N = 219)	ALS (N = 95)
	Values	*P*-value		
Number of nerves tested, n	1526		1463	549
Number of tested nerves that reported CV, n	1504		1448	498
Number of motor nerves with a CV below 80 % of the lower limit of normal, n (%)	300 (19.9 %)	<0.01 vs. NNDSP or ALS	130 (9.0 %)	40 (8.0 %)
Number of motor nerves with a CV below 70 % of the lower limit of normal, n (%)	198 (13.2 %)	<0.01 *vs*. NNDSP or ALS	97 (6.7 %)	37 (7.4 %)
Number of tested nerves that reported CMAP, n	1521		1462	542
Motor nerves with CMAP below lower limit of normal, n (%)	551 (36.2 %)	<0.01 *vs.* NNDSP or ALS	434 (29.7 %)	276 (50.9 %)
Number of tested nerves that reported F wave latency, n	1361	<0.01 *vs.* NNDSP or ALS	1234	365
Number of abnormal F latency, n (%)	844 (62.0 %)	<0.01 *vs.* NNDSP *P* > 0.05 *vs*. ALS	609 (49.4 %)	172 (47.1 %)
Number of absent F wave responses, n (%)	265 (19.5 %)	<0.01 *vs.* NNDSP or ALS	150 (12.2 %)	75 (20.5 %)
Number of motor nerves with F responses prolonged more than 120 %, n (%)	123 (9.0 %)	<0.01 *vs.* NNDSP or ALS	76 (6.2 %)	7 (1.9 %)
Number of motor nerves with F responses prolonged more than 150 %, n (%)	10 (0.7 %)		3 (0.2 %)	1 (0.3 %)
Number of tested nerves that reported distal latency, n	1521	<0.01 *vs.* NNDSP *P* > 0.05 *vs*. ALS	1463	545
Number of motor nerves with prolonged distal latency, n (%)	299 (19.7 %)		200 (13.7 %)	107 (19.6 %)
Number of motor nerves with reduced CV and prolonged c distal latencies with abnormal corresponding F responses, n (%)	171 (11.2 %)	<0.01 *vs.* NNDSP or ALS	86 (5.9 %)	37 (6.7 %)

ALS: amyotrophic lateral sclerosis; CMAP: compound muscle action potential; CV: conduction velocity; DSP: distal symmetrical polyneuropathy.

Overall P-values were obtained by comparing among diabetic DSP, non-diabetic DSP, and ALS groups.

There are more motor nerves with abnormal F latency in the diabetic DSP group compared to the ALS and non-diabetic group (*P* < 0.0001, Table 2). Furthermore, the proportion of F latencies that are prolonged more than 120 % and 150 % of upper limit of normal was significantly higher in the diabetic DSP group (9.0 % and 0.7 %) than in the non-diabetic DSP group (6.2 % and 0.2 %) and the ALS group (1.9 % and 0.3 %).

Mean DL was significantly prolonged in the diabetic DSP group compared to the non-diabetic DSP group for median, ulnar, and tibial nerves (*P* < 0.05, Table 1).

There are significantly more motor nerves with prolonged DL in the diabetic DSP group (19.7 %) compared to the non-diabetic DSP group (13.7 %, *P* < 0.0001, Table 2).

The number of absent F responses was significantly higher in the ALS (20.5 %) group compared to the diabetic DSP group (19.5 %) and non-diabetic DSP (12.2 %) groups (*P* < 0.0001, Table 2).

There are significantly more motor nerves with reduced CV and prolonged corresponding distal latencies with abnormal corresponding F responses in the diabetic DSP group (11.2 %) compared to the ALS (6.7 %) and the non-diabetic DSP (5.9 %) groups, suggesting more diffuse slowing affecting proximal, middle, and distal nerve segments in the diabetic DSP patients (*P* < 0.0001, Table 2).

The number of motor nerves with CV, DL, and F latencies fulfilling regression equations criteria for demyelination was significantly higher than the number of motor nerves fulfilling the AAN research criteria for CIDP diagnosis in all groups (Table 3). Furthermore, the number of motor nerves with CV and DL fulfilling regression equations criteria for demyelination or AAN criteria was significantly higher in the diabetic DSP group compared to non-diabetic DSP and ALS groups (Table 3).

**Table 3:** Conduction slowing in the diabetic DSP, non-diabetic DSP, and ALS groups.

Conduction velocity in demyelination range				
Groups	CV slowing in AAN range	*P* values	CV slowing in equations range	*P* values
Diabetic DSP (DDSP) (N = 1512), n (%)	256 (16.9 %)	<0.01 *vs.* NDDSP or ALS	547 (36.2 %)	<0.01 *vs.* NDDSP or ALS
Non-diabetic DSP NDDSP) (N = 1448), n (%)	36 (2.5 %)	<0.01 *vs.* ALS	329 (22.7 %)	<0.01 *vs.* ALS
ALS (N = 501), n (%)	7 (1.4 %)		47 (9.4 %)	

**Distal latency in demyelination range**				
**Groups**	**DL prolongation in AAN range**	***P* values**	**DL prolongation in equations range**	***P* values**

Diabetic DSP (N = 1522), n (%)	65 (4.3 %)	<0.01 *vs.* NDDSP or ALS	322 (21.2 %)	<0.01 *vs.* NDDSP or ALS
Non-diabetic DSP (N = 1463), n (%)	41 (2.8 %)	>0.05 *vs.* ALS	223 (15.9 %)	<0.01 *vs.* ALS
ALS (N = 546), n (%)	16 (2.9 %)		64 (11.7 %)	

**F latency in demyelination range**				
**Groups**	**F prolongation in AAN range**	***P* values**	**F prolongation in equations range**	***P* values**

Diabetic DSP (N = 1420), n (%)	348 (24.5 %)	<0.01 *vs.* NDDSP or ALS	690 (48.6 %)	<0.01 *vs.* NDDSP or ALS
Non-diabetic DSP (N = 1262), n (%)	146 (11.6 %)	<0.01 *vs.* ALS	463 (36.7 %)	<0.01 *vs.* ALS
ALS (N = 367), n (%)	58 (15.8 %)		115 (31.3 %)	

AAN: American Academy of Neurology; ALS: amyotrophic lateral sclerosis; CV: conduction velocity; DL: distal latency; DSP: distal symmetrical polyneuropathy.

NOTE: Percentage = (n/N) ∗ 100, where, n = number of nerves in the category of interest, N = number of nerves tested for that category.

Overall P-values were obtained by comparing among diabetic DSP, non-diabetic DSP, and ALS groups.

The percentage of patients having at least one motor nerve with CV slowing in the demyelinating range by AAN criteria and by regression equations ranges was significantly higher in the diabetic DSP group (32.0 % and 84.0 %) compared to non-diabetic DSP group (11.9 % and 69.4 %) and to ALS group (7.4 % and 44.2 %, Table 4).

**Table 4:** The percentage of patients having at least one motor nerve with conduction velocity (CV) slowing in the diabetic DSP (DDSP), non-diabetic DSP (NDDSP), and ALS groups.

Groups	CV slowing in AAN range	*P*	CV slowing in equations range	*P*
DDSP, N(%)	70 (32.0 %)	<0.01 *vs.* NDDSP or ALS	184 (84.0 %)	<0.01 *vs.* NDDSP or ALS
NDDSP, N (%)	26 (11.9 %)	<0.01 *vs*. ALS	152 (69.4 %)	<0.01 *vs*. ALS
ALS, N (%)	7 (7.4 %)		42 (44.2 %)	

N: number of patients.

No patients in the ALS group have more than two motor nerves with CV responses in the demyelination range either by the equations or AAN criteria. There is a significantly higher proportion of patients who have more than two motor nerves with CV responses in the demyelination range fulfilled either the equations or AAN criteria in the diabetic group compared to the axonal non-diabetic group (47 % vs 23.3 %, *P* < 0.0001). Furthermore, there are a significantly higher number of patients among those who fulfilled the above criteria had at least one motor nerve with corresponding F response in the demyelinating range fulfilling AAN criteria in the diabetic DSP group compared to the axonal non-diabetic group (30.6 % vs. 7.3 *%, P* = 0.0001).

In addition, in the diabetic subgroup among patients having at least one nerve with CV slowing in the AAN demyelination range, the estimate of likelihood to have more than 2 motor nerves with CV slowing in the demyelination range by AAN or regression equations criteria is significantly higher in the diabetic DSP group compared to non-diabetic DSP group (0.73 vs 0.52, *P* < 0.05).

The Pearson correlation coefficients, as well as slopes and intercepts from linear regression, are summarized in Table 5. The correlation coefficients suggest that there exists a significant correlation between the CMAP amplitude and CV, DL, and F responses in all studied groups.

**Table 5:** Regression analysis of amplitude-dependent variation in distal latency, conduction velocity and F response for diabetic DSP, non-diabetic DSP, and ALS groups.

Diabetic DSP Group							
	Change in DL	Change in CV	Change in F		Change in DL	Change in CV	Change in F
**Median nerve (n)**	377	372	320	**Tibial nerve (n)**	318	314	259
Intercept	2.524	2.671	2.119	Intercept	89.087	7.738	2.000
Slope	−0.248	0.208	−0.008	Slope	−0.064	1.138	−0.002
r^a^	−0.539	0.437	−0.287	r^a^	−0.431	0.437	−0.114
P-value	<0.0001	<0.0001	<0.0001	P-value	<0.0001	<0.0001	0.0084

**Non-diabetic DSP Group**							
	**Change in DL**	**Change in CV**	**Change in F**		**Change in DL**	**Change in CV**	**Change in F**

**Median nerve (n)**	336	332	282	**Tibial nerve (n)**	364	359	297
Intercept	2.381	2.777	2.083	Intercept	94.260	8.525	2.049
Slope	−0.203	0.182	−0.005	Slope	−0.087	0.881	−0.005
r^a^	−0.467	0.445	−0.187	r^a^	−0.547	0.415	−0.367
P-value	<0.0001	<0.0001	0.0126	P-value	<0.0001	<0.0001	<0.0001

**ALS Group**							
	**Change in DL**	**Change in CV**	**Change in F**		**Change in DL**	**Change in CV**	**Change in F**

**Median nerve (n)**	122	121	62	**Tibial nerve (n)**	135	104	88
Intercept	2.175	2.925	2.102	Intercept	97.573	8.926	2.020
Slope	−0.133	0.141	−0.008	Slope	−0.071	0.767	−0.004
r^a^	−0.601	0.494	−0.357	r^a^	−0.352	0.369	−0.362
P-value	<0.0001	<0.0001	0.004	P-value	<0.0001	0.0001	0.005

NOTE: Only Median and Tibial nerve data is shown. CV, conduction velocity; DL, distal latency. n: number of motor nerves.

a Pearson correlation coefficient.

**Fig. 1:**
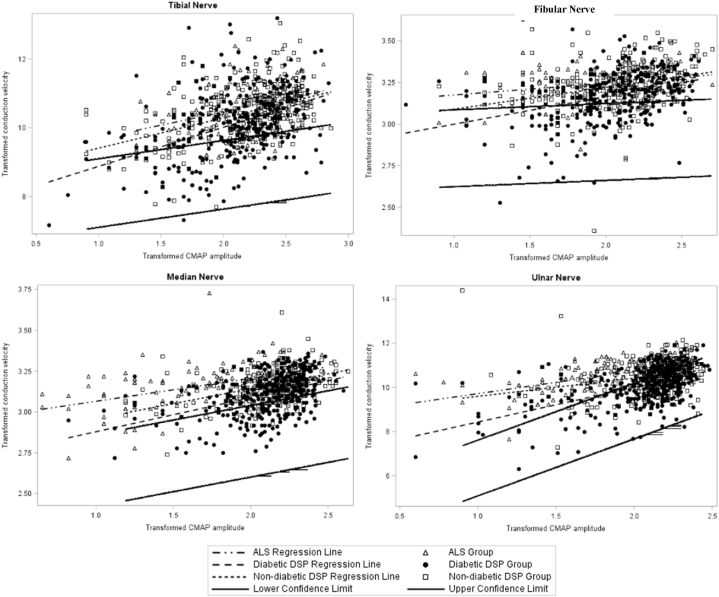
**Regression plots of normalized CV versus normalized CMAP amplitude for diabetic DSP, non-diabetic DSP and ALS groups.** Regression plots of converted normalized CV (y-axis, dependent variable) and converted normalized CMAP amplitude (x-axis, independent variable) are illustrated for diabetic DSP, non-diabetic DSP and ALS groups. All raw CMAP amplitudes and CV data were expressed as fractions of the lower limit of normal then transformed to Log_10_ for CMAP amplitude, square root for CV of the tibial and ulnar nerves, fourth root for CV of median and fibular nerves to achieve a linear relationship between CMAP amplitude and CV measurements. Additional CMAP amplitude-independent CV slowing was observed in all motor nerves in the diabetic DSP relative to non-diabetic DSP and ALS groups: the plot demonstrated significant correlation between CMAP amplitude and CV in all diabetic DSP and non-diabetic studied motor nerves. The y-intercept of regression was significantly lower in the diabetic DSP group compared to the non-diabetic DSP group and to the ALS group, which cannot be explained by the difference in the slopes of these groups. Diabetic DSP data points are depicted by closed triangles, non-diabetic DSP data points are depicted by open triangles, and ALS data are depicted by open circles. Regression lines for diabetic DSP, non-diabetic DSP, and ALS are depicted by a solid bold line, a solid line, and a broken line respectively.

In addition, analysis of covariance was conducted to assess the association of CMAP amplitude (as an independent variable) with CV (as dependent variables) for the 3 groups. The y-intercept of the regression line was significantly lower in the diabetic DSP group compared to the non-diabetic DSP ALS groups, which cannot be explained by the difference in the slopes of these groups (*P* < 0.05, Fig. 1).

There is a significant difference in the slopes of the CV for the median, fibular, and ulnar nerves, but not for the tibial nerve, between the diabetic DSP group and the ALS group (*p* < 0.05, Fig. 1). Similar findings were observed for the ulnar nerve in the diabetic DSP group compared to the non-diabetic DSP group (*P* < 0.05). These findings suggest that the corresponding CV for the same CMAP amplitude is significantly lower in the diabetic group compared to the ALS group in median, fibular, and ulnar nerves, and significantly lower compared to the non-diabetic DSP group for the ulnar nerve.

## 4 Discussion

Our study demonstrated that a high proportion of patients in the diabetic DSP group has at least one motor nerve with CV slowing in the equation's demyelinating range, compared to the axonal non-diabetic and to the ALS groups. In addition, there is a significant increase in the likelihood of having more than two motor nerves with conduction slowing in the AAN or regression equations confidence intervals in the diabetic DSP subgroup with at least one motor nerve with CV slowing in the AAN demyelination range, compared to the ALS and to the non-diabetic neuropathy groups.

The findings suggest that the conduction slowing in diabetic DSP is beyond what is expected from a pure axonal loss and that primary or secondary demyelination is contributing to the electrodiagnostic findings seen in these patients. Moreover, the presence of at least one motor nerve with CV in the AAN demyelinating range increased the likelihood of having other motor nerves with abnormal conduction slowing beyond what is expected from a pure axonal loss. This is also supported by the fact that the coefficient for the slope of CMAP amplitude compared to CV in the multiple linear regression analyses was significantly different between the diabetic DSP and ALS groups. CV of all studied nerves was demonstrated to be lower in the diabetic group compared to the ALS group. In addition, there are lower y-intercepts of CV in all studied nerves in the diabetic DSP group compared to ALS and axonal non-diabetic groups.

To differentiate between CIDP, a potentially treatable disorder, and axonal polyneuropathy with loss of fast-conducting axons, an irreversible process, the AAN set research electrodiagnostic criteria with high specificity and low sensitivity [17]. Conduction slowing that seldom fulfills the AAN criteria for CIDP has been observed in diabetic DSP in several studies [[19], [20], [21], [22], [23]]. Although a significant difference in conduction slowing was found between diabetic DSP patients and non-diabetic purely axonal neuropathies, there was substantial overlap between the electrophysiologic findings in the two groups [10,11]. This overlap is caused by the difficulties in documenting demyelination in diabetic DSP, especially in mild or early cases in which sufficient demyelination has not yet occurred, in advanced cases in which secondary axon loss precludes detection of demyelination, and in diabetic patients with demyelination confined mainly to proximal nerve segments that are difficult to study with conventional electrodiagnostic testing. To improve the sensitivity of the conventional electrophysiologic criteria for differentiating demyelination from axonal neuropathy, several studies have analyzed the relationship between CV slowing and CMAP amplitude for determination of CMAP-dependent CV slowing (slowing related to loss of fast conduction axons) versus CMAP-independent CV slowing (slowing beyond that which is expected from pure axonal loss) [[9], [10], [11]].

Additionally, we demonstrated that regression equations confidence intervals derived from a well-defined cohort of patients with CIDP, are more sensitive than AAN criteria in identifying conduction slowing beyond what is expected from pure axonal loss. Our results in this study are consistent with previous investigations [9,13].

The regression equations developed in this study were defined for each CMAP amplitude, and a confidence interval for conduction slowing was related to a primary demyelinating process. When these regression equations were applied to electrodiagnostic data obtained from diabetic DSP, non-diabetic DSP, and ALS groups, a significantly different coefficient for the slope of CMAP versus CV and lower y-intercept of CV was observed in the diabetic DSP group compared to the other two groups, supporting CV slowing unrelated to a pure axonal loss. When the regression equations were applied to electrodiagnostic data from the ALS group, no patients were found to have more than two motor nerves with CV slowing in the AAN or equations ranges. Therefore, setting up a minimum number of motor nerves for each diabetic DSP patient with CV slowing fulfilling the regression equations should be considered. In addition, the likelihood of having more than two motor nerves with conduction slowing as determined by AAN criteria or regression equations criteria is significantly higher in the diabetic DSP group with at least one nerve with CV slowing in the AAN criteria compared to non-diabetic DSP, supporting the probable presence of demyelination in the diabetes group. However, conventional electrodiagnostic testing results as well AAN criteria do not suggest demyelination when the process is mild.

With the absence of specific serum biomarkers for CIDP, the use of our regression equations in diabetic DSP patients may improve the ability to identify mild demyelination in diabetic DSP. Since the regression equations were designed to achieve the best linear relationship between CMAP amplitude and the corresponding CV, the resulting confidence interval may not capture severe CV in the AAN demyelinating range. Therefore, combining the regression equations with the AAN criteria for demyelination and an adequate clinical assessment may improve the sensitivity and specificity of the regression equations to better identify conduction slowing that is not resulting from a pure axonal loss and could potentially be amenable to treatment.

The study used CIDP for regression analysis may have some limitations. Demyelination in CIDP may occur in a multifocal pattern [24]; for example, nerve conduction studies have shown that demyelination is present in some but not in all nerves and nerve segments [25,26]. The regression equation calculated by CIDP may also be influenced by secondary axonal degeneration and conduction slowing in CIDP may be influenced by axonal polyneuropathies [27]. To overcome the shortcoming, further study should perform the regression analysis in specified demyelinating nerves in diabetic neuropathy, which is not so easy to accomplish in the clinical setting.

## 5 Conclusion

We demonstrated that regression analysis of CMAP amplitude versus motor nerve conduction study parameters identifies conduction slowing in diabetic DSP patients beyond what is expected from axonal loss observed in ALS and axonal non-diabetic DSP groups. Our regression analysis is more sensitive than conventional electrodiagnostic testing to detect conduction slowing compatible with the diagnosis of mild demyelination. As there is some overlap between conduction slowing from axonal loss and mild demyelination not captured by regression analysis, and also given the lack of specific biomarker for acquired demyelination, combining regression equations with an adequate clinical evaluation and the AAN criteria may improve the sensitivity and specificity to identify demyelination in diabetic DSP.

## CRediT authorship contribution statement

**Nizar Souayah:** Writing – original draft, Supervision, Funding acquisition, Conceptualization. **Zhao Zhong Chong:** Writing – review & editing, Project administration, Data curation. **Tejas Patel:** Investigation, Formal analysis. **Abu Nasar:** Investigation, Formal analysis. **Ankit Pahwa:** Methodology, Formal analysis, Data curation. **Howard W Sander:** Writing – review & editing, Formal analysis.

## Data availability

The data associated with this study have not been deposited into any public repository. The data included supporting this article is available from the corresponding author upon reasonable and justified request.

## Declaration of competing interest

The authors declare that they have no known competing financial interests or personal relationships that could have appeared to influence the work reported in this paper.
